# Baseline gene signatures of reactogenicity to Ebola vaccination: a machine learning approach across multiple cohorts

**DOI:** 10.3389/fimmu.2023.1259197

**Published:** 2023-11-08

**Authors:** Patrícia Conceição Gonzalez Dias Carvalho, Thiago Dominguez Crespo Hirata, Leandro Yukio Mano Alves, Isabelle Franco Moscardini, Ana Paula Barbosa do Nascimento, André G. Costa-Martins, Sara Sorgi, Ali M. Harandi, Daniela M. Ferreira, Eleonora Vianello, Mariëlle C. Haks, Tom H. M. Ottenhoff, Francesco Santoro, Paola Martinez-Murillo, Angela Huttner, Claire-Anne Siegrist, Donata Medaglini, Helder I. Nakaya

**Affiliations:** ^1^ Oxford Vaccine Group, University of Oxford, Oxford, United Kingdom; ^2^ Department of Clinical Sciences, Liverpool School of Tropical Medicine, Liverpool, United Kingdom; ^3^ Department of Clinical and Toxicological Analyses, School of Pharmaceutical Sciences, University of São Paulo, São Paulo, Brazil; ^4^ Microbiotec Srl, Siena, Italy; ^5^ Division of Infectious Diseases, Cincinnati Children’s Hospital Medical Center, Cincinnati, OH, United States; ^6^ Artificial Intelligence and Analytics Department, Institute for Technological Research, São Paulo, Brazil; ^7^ Laboratory of Molecular Microbiology and Biotechnology (LAMMB), Department of Medical Biotechnologies, University of Siena, Siena, Italy; ^8^ Department of Microbiology and Immunology, Institute of Biomedicine, Sahlgrenska Academy, University of Gothenburg, Gothenburg, Sweden; ^9^ Vaccine Evaluation Center, BC Children’s Hospital Research Institute, University of British Columbia, Vancouver, BC, Canada; ^10^ Department of Infectious Diseases, Leiden University Medical Center, Leiden, Netherlands; ^11^ Centre for Vaccinology, Faculty of Medicine, University of Geneva, Geneva, Switzerland; ^12^ Infectious Diseases Service, Geneva University Hospitals, Geneva, Switzerland; ^13^ Department of Medical Biotechnologies, University of Siena, Siena, Italy; ^14^ Scientific Platform Pasteur-University of São Paulo, São Paulo, Brazil; ^15^ Hospital Israelita Albert Einstein, São Paulo, Brazil

**Keywords:** Ebola, rVSVDG-ZEBOV-GP vaccine, baseline gene signatures, adverse events, vaccine safety, personalized vaccinology, machine learning, data integration

## Abstract

**Introduction:**

The rVSVDG-ZEBOV-GP (Ervebo®) vaccine is both immunogenic and protective against Ebola. However, the vaccine can cause a broad range of transient adverse reactions, from headache to arthritis. Identifying baseline reactogenicity signatures can advance personalized vaccinology and increase our understanding of the molecular factors associated with such adverse events.

**Methods:**

In this study, we developed a machine learning approach to integrate prevaccination gene expression data with adverse events that occurred within 14 days post-vaccination.

**Results and Discussion:**

We analyzed the expression of 144 genes across 343 blood samples collected from participants of 4 phase I clinical trial cohorts: Switzerland, USA, Gabon, and Kenya. Our machine learning approach revealed 22 key genes associated with adverse events such as local reactions, fatigue, headache, myalgia, fever, chills, arthralgia, nausea, and arthritis, providing insights into potential biological mechanisms linked to vaccine reactogenicity.

## 1 Introduction

Ebola virus disease (EVD) is a severe and fatal infectious disease (1). rVSVΔG-ZEBOV-GP, under the name of Ervebo®, is given as a single-dose vaccine. It is a recombinant vaccine against the live and attenuated vesicular stomatitis (VSV) virus, in which the gene encoding for the VSV envelope glycoprotein has been replaced by the Ebola strain Zaire virus (ZEBOV-GP) glycoprotein gene (2). This vaccine is highly immunogenic for at least two years (3).

Live replicating VSV-based vaccines can elicit potent humoral (4–6) and strong cellular immune responses against viral (7–9). However, replication-competent vectors are frequently associated with a higher risk for adverse events (AE) (10). Although rVSVΔG-ZEBOV-GP is safe, immunogenic, and protective in human trials (11), vaccinees may report transient adverse reactions such as fever, inflammation, arthritis, dermatitis and vasculitis (11–14). Vaccine viraemia is common and associated with frequent mild-to-moderate acute inflammatory reactions and, in some vaccinees, viral dissemination, leading to arthritis and occasional dermatitis (13, 15). The occurrence of arthritis, arthralgia and other forms of joint swellings and tissue infiltration was higher in European and US vaccinees than in participants from Africa. Arthritis cases have been reported in approximately 23% (24 – 102) of vaccinees from Switzerland (11) and 4.5% (19 - 418) from USA (16), whereas a low incidence of 2.5% (1 - 40) (12) or non-incidence has been reported in Kenya and Gabon, respectively (12). The cases occurred mainly in participants aged 40 years and above, and they were self-limiting with no sequelae (15).

Although these AE did not prevent vaccine uptake (15), identifying baseline reactogenicity signatures represents an important step toward the development of personalized vaccinology and could enhance public confidence in the safety of vaccines (17). Recent studies have reported baseline predictors of post-vaccination responses for human influenza virus (18, 19), hepatitis B virus (20), as well as malaria (21) vaccination. Nonetheless, few studies focused on reactogenicity (22, 23).

In this study, we report a machine learning (ML) approach to unravel multicohort baseline transcriptional reactogenicity signatures to rVSVΔG-ZEBOV-GP. We have integrated AE reported by participants from Switzerland, USA, Gabon and Kenya clinical trials with the expression of 144 genes before the administration of rVSVΔG-ZEBOV-GP. We have identified an AE signature in which twenty-two genes and nine adverse events appear to be associated. Crucially, despite the varying baseline, the genes contribute to predicting delineated stable baseline differences across cohorts, raising the prospect of screening for AE propensity before vaccination.

## 2 Methods

### 2.1 Study design and ethics statement

The data was obtained from four clinical trials conducted for the VSV-EBOVAC and VSV-EBOPLUS Consortia on 3 different continents: North America (Phase I, randomized, double-blind, placebo-controlled, dose-response trial in the USA; Registration number NCT02314923), Europe (Phase I/II, randomized, double-blind, placebo-controlled, dose-finding trial in Geneva, Switzerland; Registration number NCT02287480) and Africa (Phase I, randomized, open-label, dose-escalation trial in Lambaréné, Gabon, and a phase I, open-label, dose-escalation trial in Kilifi, Kenya; Registration numbers PACTR201411000919191 and NCT02296983, respectively).

The trial protocols were reviewed and approved by the WHO’s Ethics Committee as well as by local ethics committees (USA trial: the Chesapeake Institutional Review Boards (Columbia, MD, USA) and the Crescent City Institutional Review Board (New Orleans, LA, USA); Geneva trial: the Geneva Cantonal Ethics Commission and the Swiss Agency for Therapeutic Products (Swissmedic); Lambaréné trial: the Scientific Review Committee of Centre de Recherches Médicales de Lambaréné (CERMEL), the Institutional Ethics Committee of CERMEL, the National Ethics Committee of Gabon, and the Institutional Ethics Committee of the Universitätsklinikum Tübingen; Kilifi trial: Kilifi Ethics Committee). Placebo recipients received a normal saline injection. Information about randomization and masking and vaccine procedures were published elsewhere (16).

### 2.2 Available data from VSV-EBOVAC and VSV-EBOPLUS

Reactogenicity data from 782 healthy adult volunteers were collected: 512 from the United States of America (418 vaccinated and 94 placebo recipients), 115 from Geneva, Switzerland (102 vaccinated and 13 placebo recipients), 115 from Lambaréné, Gabon, and 40 from Kilifi, Kenya.

Peripheral whole blood samples were collected at several time points for transcriptomic evaluation (115 in Switzerland, 144 in the USA, 83 in Gabon, and 39 in Kenya). However, since the interest of this work was to study the host’s aptitude to develop adverse reactions, we have used only expression data obtained at day 0, before immunization. Among the adults included in the study, 343/782 (43.9%) volunteers from the four cohorts had the expression of 144 genes quantified from a multiplex RT-PCR quantitative platform, which has been amplified using a two-color ligation-dependent probe called dcRT-MLPA (24), and was previously published (25).

### 2.3 Outcomes

We performed a predictive reactogenicity cohort evaluation within the phase 1 trials from Switzerland (randomized), Gabon (dose-escalation), Kenya, and USA. The biological and clinical outcomes of these studies have been reported elsewhere (3, 12, 16, 26).

Reactogenicity data were collected until day 14, day 28 and day 365 in the American, African, and European cohorts, respectively. For all cohorts (USA, Switzerland, Gabon and Kenya), we have selected the AEs of grade 1, 2 or 3 (mild, moderate or severe, respectively) as published previously for our VSV-EBOVAC consortia partners (3, 12, 16, 26).

**Figure 1:**
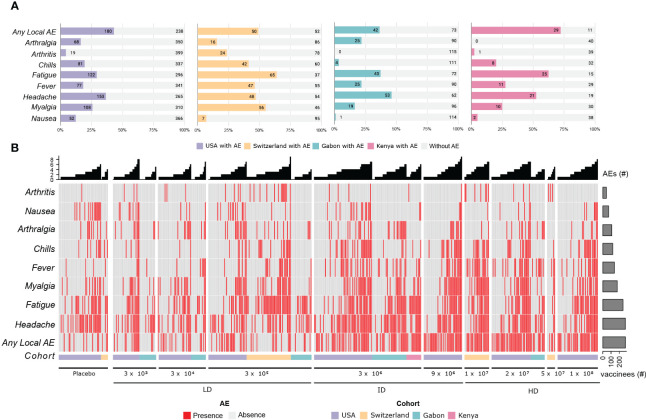
Adverse Events description for the 4 cohorts. **(A)** The stacked bar plots shows the absolute number and the frequency of the main adverse effects described in the first 14 days after vaccination with rVSVΔG-ZEBOV-GP, in the cohorts. The colored portion of each bar represents the number of participants who reported each of the adverse events, with the cohorts being represented by the colors purple (USA), yellow (Switzerland), green (Gabon), pink (Kenya) and light gray (no adverse events reported). **(B)** Heatmap showing the presence (red) and absence (light gray) of the most important adverse events. The most frequent AEs are shown at the bottom of the heatmap, and the columns are ordered per dose and cohort, as shown in the bottom annotation (USA, purple; Switzerland, yellow; Gabon, green and Kenya, pink). The number of AEs per participant is shown in the bar plot at the top, and the total number of reported adverse events is shown in the bar plot on the right.

Adverse event terms were standardized across all four cohorts. For the USA cohort, the terms “tenderness” and “pain in extremity” and for Switzerland, Kenya, and Gabon, the term “pain at site” were considered “any local AE”. The term pyrexia in the USA and “subjective fever” in Switzerland, Kenya and Gabon were considered “fever”. AE terms reported by less than 5% of the participants were removed from the next analysis. The following is the final list of adverse events in the order of frequency: “any local AE”, “headache”, “fatigue”, “myalgia”, “fever”, “chills”, “arthralgia”, “nausea”, “arthritis”. The incidence within each cohort is shown in **Figure 1**.

### 2.4 Gene expression profiling

The human transcriptomic profiles of the response to the rVSVΔG-ZEBOV-GP vaccine were evaluated by the quantitative multiplex platform RT-PCR, which performs amplification using a two-color ligation-dependent probe (dcRT-MLPA). PAXgene blood RNA tubes (PreAnalytiX, Hombrechtikon, Switzerland) with 2.5 ml venous blood were collected and stored at -80°C. RNA isolation was performed using the PAXgene blood miRNA kit (PreAnalytiX) according to the manufacturer’s automated protocol, including on-column DNase digestion. RNA yield was quantified using an RNA Broad Range assay Kit (ThermoFisher) with a Qubit fluorometer (ThermoFisher, Wilmington, DE, USA). The dcRT-MLPA (MLPA) assay accounts for 144 genes of critical importance whose involvement in innate and adaptive immune responses (24) is documented and used to determine the gene expression profiles of people vaccinated with rVSVΔG-ZEBOV-GP. The gene expression values thus generated were normalized according to the expression of the housekeeping gene GAPDH and transformed to log2, whereas the quality control was performed as described in previous works (25, 26). The function removeBatchEffect from limma package in R was used to remove the batch effect of the dcRT-MLPA plaque within each cohort.

### 2.5 Statistical analysis

GAPDH-normalized log2-transformed gene expression levels at baseline for each cohort were used for integrative analysis. Gene expression comparisons were conducted between volunteers with and without adverse events within each cohort and when combining all the cohorts. The non-parametric Wilcoxon test with Benjamini-Hochberg correction for multiple testing was applied for statistical significance. An adjusted P-value (q-value) of less than 0.05 was set as the threshold for identifying significant genes for the comparison of groups with or without adverse events. Analyses were performed with R software (version 4.0.4).

### 2.6 Feature selection machine-learning-based approach

**Figure 2:**
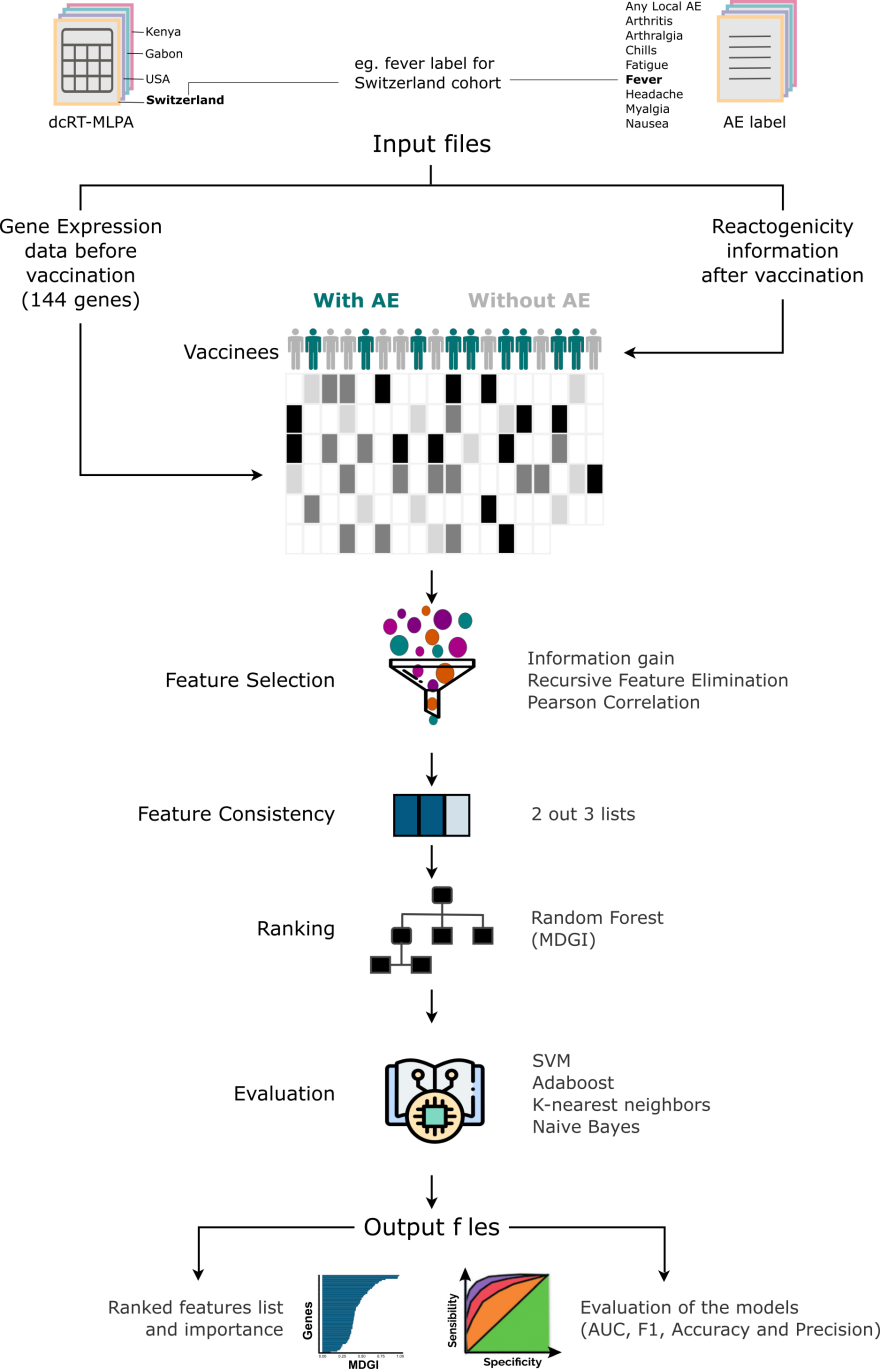
Machine-learning-based Algorithm description performed for each adverse effect per cohort. The score measures the ability of a feature to distinguish the outcome groups. First, considering that the quality of the predictive models depends on the quality of features used, the method performs the selection of features. The selection is based on the combination of 3 different methods: Pearson’s correlation, Kbest and Recursive Feature Elimination (RFE). After generating a list of features for each method, a unique list is generated by selecting features from the intersection of 2 out of 3 methods. From this list, the method generates the ranking importance obtained from the Random Forest model and removes features with an importance value equal to 0. Subsequently, it evaluates the quality of gene list in discriminating the adverse events (AEs) classes in 4 machine learning models trained with the algorithms Support Vector Machine (SVM), k-Nearest Neighbors (kNN), Naive Bayes and AdaBoost Classifier. Thereafter, the tool generates a table with the F1-score, Area Under the curve (AUC), the accuracy and precision values obtained from each model with the selected features, and the median and harmonic mean calculated from all methods and metrics.

The expression of the 144 immune-related genes on Day 0 (before immunization) and the information of reactogenicity obtained after immunization, for the volunteers of each cohort, were used as input files. Then, our algorithm which is a robust ML-based feature prioritization tool fully described in **Figure 2** was run.

To summarize, our method first performs feature selection using three different methods: Pearson’s correlation, Kbest and Recursive Feature Elimination (RFE). After generating a list of features for each method, a unified list is produced by selecting features from the intersection of 2 out the 3 methods. From this list, our approach orders the list using the Mean Decrease Gini Index (MDGI) obtained with the function ‘feature_importances_’ from the model trained with the Random Forest algorithm implemented in scikit-learn as “RandomForestClassifier”. The features with an importance value equal to 0 are removed. Finally, it assesses the discriminatory power of the selected features and determine their effectiveness in classifying the different groups by using models trained with various machine-learning algorithms such as: Support Vector Machine (SVM), k-Nearest Neighbors (kNN), Naive Bayes and AdaBoost Classifier. Thereafter, the tool generates as output a table with the values of F1-score, area under the curve (AUC), accuracy, and precision, obtained from each model with the selected features. Using the machine-learning-based approach, we assessed the importance of genes in classifying volunteer groups with or without the selected AEs (frequency > 5%), which are “any local AE”, “headache”, “fatigue”, “myalgia”, “fever”, “chills”, “arthralgia”, “nausea”, “arthritis”. The Support Vector Machine (SVM), k-Nearest Neighbors (kNN), Naive Bayes, AdaBoost Classifier and Random Forest ML algorithms were trained with a 10-fold cross-validation classification method. All the analyses were performed in Python (version 3.8.11). The library scikit-learn 0.24.2 was used for training the ML algorithms. All the hyperparameters were defined as default. A more detailed description of the ML-based feature prioritization tool can be found on the extended methods in the **Supplementary Material**.

### 2.7 Network construction

**Figure 3:**
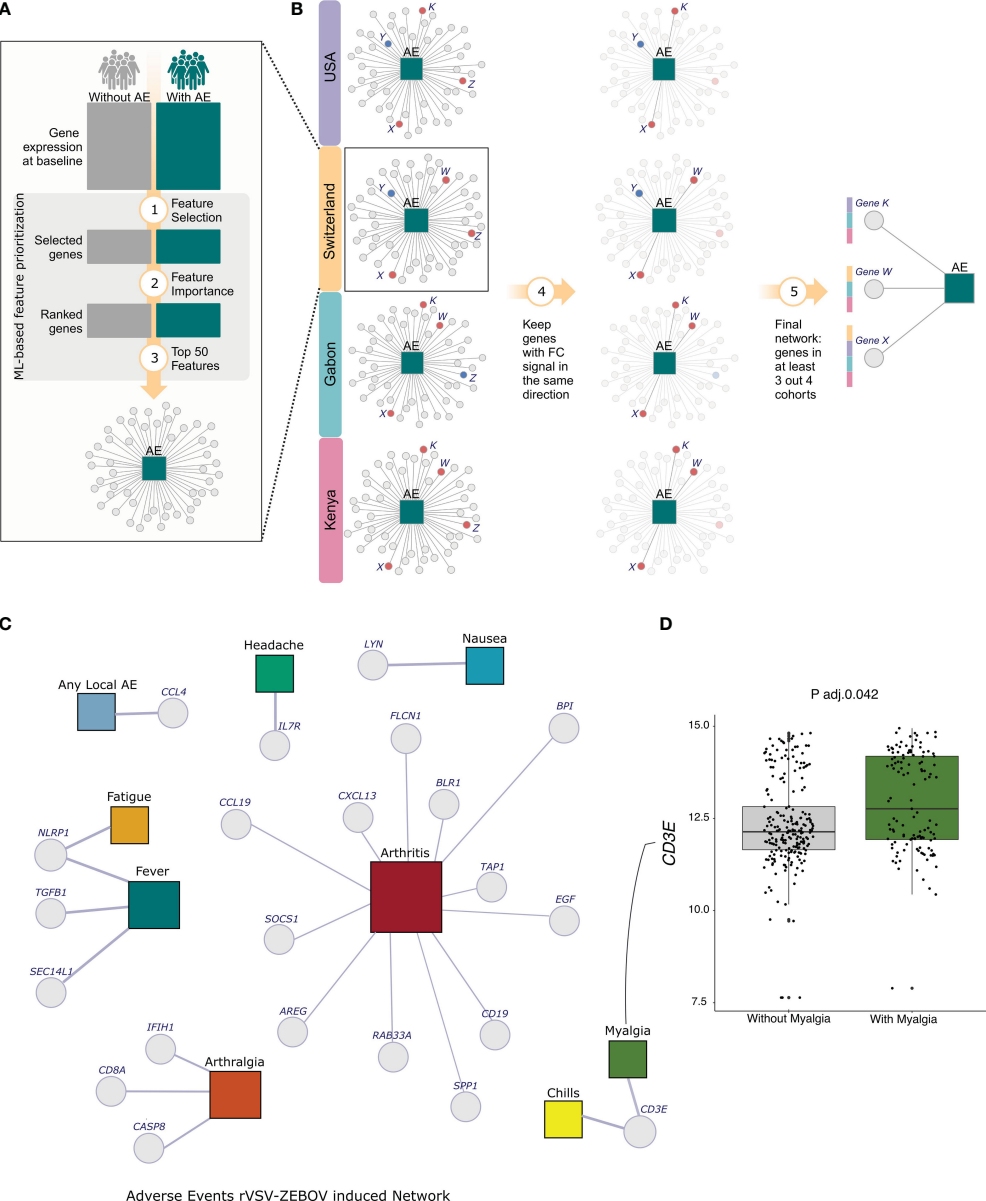
Analysis Scheme and Network of selected genes for all 9 Adverse Events. **(A)** The dcRT-MLPA with the expression of 144 genes per cohort was used to select the best features for classifying participants with or without each one of the adverse events individually. The machine-learning-based method was used for feature selection and ordering. **(B)** Among the selected ordered features, the top 50 from each cohort were chosen, and those with consistent fold-change signs across all four cohorts and shared by more than 50% of cohorts were kept. **(C)** Network Adverse Events description for the 4 cohorts. The adverse events are represented by colored squares, and the genes are represented by Light-grey circles. The squares representations are as follows: Light-blue - any local AE, Green - headache, Blue - nausea, Dark-Red - arthritis, Dark-green - myalgia, Light-yellow - chills, Orange - arthralgia, Surfie-Green - fever and Dark-yellow - fatigue. **(D)** The boxplot shows the log2 transformed expression of the gene EGF in Arthritis and non-arthritis participants.

From the list of ranking of importance (MDGI), the top 50 features from the list were selected (**Figure 3A**) and their consistency across the four cohorts were evaluated. The genes with the same fold-change direction in 100% of the cohorts and shared by more than 50% of the cohorts (3 out 4, 3 out 3, 2 out 2) are kept (**Figure 3B**). Finally, we integrated the genes with the AEs in a network constructed using Gephi software (27) (**Figure 3C**).

## 3 Results

### 3.1 Reactogenicity was frequent but generally mild

The vaccine proved to be safe, even if associated with transient reactogenicity (11). We observed injection-site, systemic reactogenicity and medication use for 7 days after injection and at follow-up timepoints (days 14 and 28). We collected reactogenicity information from a total of 782 participants: 115 from the Swiss cohort (102 vaccinated and 13 placebo), 115 from Gabon, and 40 from Kenya. The remaining 512 participants were from the United States (418 vaccinated and 94 placebo). The participants included 488 males and 355 females, and the median age was 35 years (18-63), the sex and age median per cohort is shown in the **Supplementary Tables 1** and **2**, respectively.

Solicited and unsolicited adverse events were frequent. Majority of participants reported adverse effects in the first 14 days after vaccination, mostly mild and moderate. The side effects induced by rVSVΔG-ZEBOV-GP vaccination are self-limiting and relatively mild (**Supplementary Figure 1**). The most frequent side effects observed were any local AE (53.25%), fatigue (49.17%), headache (46.55%), myalgia (31.27%), fever (28.90%), chills (21.50%), arthralgia (13.67%), nausea (6.30%) and arthritis (6.39%).

Several people reported AE grade 1, including placebo recipients. Of the 782 participants, 638 (81.6%) have reported at least one adverse event, with the majority being mild or moderate. At least one adverse event (grade 1, 2 or 3) was reported by 328 of 418 (78.47%) vaccinees from the US cohort and by 96 of 102 (94.1%) from the Swiss cohort. In African cohorts, 97 of 115 (84.3%) and 37 of 40 (92.5%) participants from Gabon and Kenya, respectively, reported adverse events. Whilst among the placebo recipients, 69 of 98 (70.4%) and 11 of 13 (84.6%) participants have reported adverse events in the US and the Swiss cohorts, respectively. Grade 3 symptoms were reported by 4 of 40 (10%) vaccinees from Kenya, 23 of 418 (5.5%) from the USA, and 11 of 102 (10.8%) from Switzerland; none were reported in Gabon. Arthritis was reported in 24 participants (≃ 23%) from the Swiss cohorts, 1 (2.5%) from Kenya and 19 (≃ 4.5%) from the United States.

The complete information of adverse events, before the nomenclature combination and filtering, is shown in **Supplementary Figure 1**. The onset differs mainly for the grade 3 AEs (**Supplementary Figure 1B**). In general, the percentage of vaccinees reporting AE grade 2 or 3 increases in higher doses (**Supplementary Figure 1C**).

### 3.2 Associations between gene expression and adverse events

We collected dcRT-MLPA and reactogenicity data for a total of 343 vaccinees. **Supplementary Figure 2** describes the number and proportion of volunteers who have dcRT-MLPA data (**Supplementary Figure 2A**), as well as the number of vaccinees who had reported the presence or absence of each of the adverse events per cohort (**Supplementary Figure 2B**). The percentage of participants with dcRT-MLPA available per cohort and the comparison of the ranking and frequency of adverse effects between all participants with reactogenicity data and the participants with available dcRT-MLPA data are shown in the **Supplementary Tables 3** and **4**, respectively.

Considering only participants with available dcRT-MLPA data, local AE is the most frequent (48.4%), followed by fatigue (48.1%), headache (47.5%), myalgia (36.7%), fever (34.4%), chills (29.9%), arthralgia (17.2%), arthritis (8.2%) and nausea (7%).

### 3.3 Feature selection per cohort and adverse event using our machine-learning-based approach

We integrated the reactogenicity data with the available expression data to understand the propensity of populations to AEs induced by vaccination with rVSVΔG-ZEBOV-GP. For this, we ran our ML-based feature prioritization tool described in **Figure 2**.

### 3.4 Multicohort baseline transcriptional-reactogenicity network

We kept the top fifty genes selected using our machine-learning-based approach (**Figure 3A**), keeping only the genes with the same fold-change signal across all cohorts. Next, we filtered out those shared by more than 50% of the cohorts (3 out 4, 3 out 3, 2 out 2) approach (**Figure 3B**). Finally, we integrated the genes with the AEs in a network constructed using Gephi software (27). The size of the nodes represents the degree, which denotes the number of connections in the network (**Figure 3C**).

After the integration, we selected a total of 22 genes for 9 adverse events. Interestingly, for six adverse events, only one gene was selected (**Figure 3C**). We selected the genes *CCL4* and *IL7R*, which are regulatory T-cell-associated markers, for local AE and headache, respectively. Both genes exhibited an increased expression in volunteers with adverse events, though it was not statistically significant. Nausea was associated with the gene *LYN*, which encodes a tyrosine kinase. For fatigue, we only selected the gene *NLRP1*, known to be a key mediator of programmed cell death. We selected the same gene for fever, but in combination with the genes *TGFB* and *SEC14L1*. Although the expression of the *NLRP1* gene increased in participants with both adverse events, the levels of this gene were significant only in the comparison of participants with or without fatigue (Adj. p-value = 0.0022). Similarly, we selected the gene *NLRP1* for two adverse events, and the gene *CD3E*, a T-cell marker, for chills and myalgia. However, only myalgia participants showed a significant increase in *CD3E* gene (**Figure 3D**, Adj. p-value 0.0409).

In the classification of participants with or without arthralgia, three genes were selected, namely *CASP8* (apoptosis-related genes), *CD8A* (Marker of lymphocyte subsets) and *IFIH1* (innate immune response related gene). This result was consistent across three cohorts since no arthralgia cases were reported in the Kenya cohort.

We identified a total of 12 genes that are associated with arthritis classification. It’s important to note that the relatively high number of genes may be attributed to the fact that we only considered cohorts from Switzerland and USA since Gabon and Kenya had a lack of arthritis cases.

The arthritis-associated genes are *BLR*, G protein-coupled receptor; *RAB33A*, Small GTPases - (Rho) GTPase activating proteins; the chemokine gene *CCL19*; the cell growth associated genes *AREG* and *EGF*; the B cell marker gene *CD19*; the tumor suppressor gene *FLCN1*; *BPI*, which is associated with anti-microbial activity; *SPP1*, an epithelial-mesenchymal transition and Inflammation marker; and innate immune responses related genes, *CXCL13*, *SOCS1* and *TAP1*—the first is a myeloid associated gene, whilst the last two are IFN signaling genes.

Among the arthritis-associated genes *AREG*, *BPI, EGF*, *FLCN1*, *RAB33A*, *SOCS1*, *SPP1* and *TAP1* have a significant difference between arthritis and non-arthritis volunteers. Among them, *AREG*, *BPI* and *TAP1* genes showed an increased expression in arthritis participants.

## 4 Discussion

Although many studies describe the reactogenicity of the vaccine, only few define reactogenicity signatures. The majority of them focus on cytokines, as it has long been assumed that vaccine reactogenicity is reflected in innate responses and inflammation (28). Moreover, studies describing reactogenicity signatures using expression data are even rarer (25). To the best of our knowledge, this is the first method that integrates baseline gene expression data with several vaccine-induced reactogenicity across 4 cohorts.

The most onerous challenge in baseline data analysis is dealing with batch effects. In multi-cohort studies, data variability can be caused by inter-subject variation, technical discrepancies from sample collection or/and data acquisition and processing. In addition, the subset of participants with available expression data may not adequately represent the overall population, which raises concerns about generalization. Much like the expression data, the number of adverse events also varies within the cohort. Participants from Switzerland reported higher rates of adverse events in comparison with the other sites. Several factors may contribute to this disparity, including differences in reporting practices and clinical investigation approaches. These events, often of mild or moderate severity and not easily attributed to vaccination, may go unreported. Additionally, variations in host factors that regulate inflammatory and immune responses, such as age, sex, fitness level, physical activity, body-mass index, baseline immunity, and human leukocyte antigen types, likely differ between study populations (11, 26). Associations between vaccine dose, innate responses, and reactogenicity was already shown by our collaborators (26). The frequency of self-reported, vaccine-induced AEs is notably lower in African settings. This same pattern was reported by Muyanja and colleagues (2014), when immunizing volunteers from Uganda and Switzerland with the yellow fever vaccine 17D (YF-17D) (29). Although self-reported vaccine-induced adverse events are notably less frequent in African settings, a study from Huttner and collaborators (2017) reveals that this reduced incidence does not correlate with weaker innate responses or higher baseline concentrations of anti-inflammatory cytokines like IL-10. Innate responses were found to be similar between European and African volunteers (26). This finding emphasizes the importance of assessing vaccine safety in the settings where they will be used (26). This is the reason we have analyzed each cohort individually before integrating the results for consistency.

Here, a baseline consistent vaccine reactogenicity signature was found across 4 different cohorts from 3 different continents. The signatures came from the gene expression analysis of 144 genes at baseline (before injection) from volunteers who had received recombinant vesicular stomatitis virus-vectored Zaire Ebola vaccine. Following which we were able to associate 22 genes with the following adverse events: any local AE, fatigue, headache, myalgia, fever, chills, arthralgia, nausea, and arthritis.

Interestingly, regulatory T-cell markers were associated with the most frequent adverse events. The genes *CCL4* and *IL7R* were associated with local AE and headache, respectively. *CCL4* and other cytokines, such as *CCL2, CCL5 and CCL8*, have been associated with the recruitment of neutrophils, eosinophils, more monocytes and DCs to the injection site in response to the activation of myeloid cells after MF59 adjuvant administration (30). The same marker levels in lymph nodes and in the muscle at the injection site has strong positive correlation in a study that evaluated mice immunized with four licensed vaccines (31). Similarly, after injection of mRNA vaccine, a strong production of chemokines (including CCL4) at the site of injection was observed by Kowalczyk and colleagues (2014) (32). Hence, the high volume of cells in the injection site can be related to local reactions.

Headache was the most frequent adverse event in a Phase Ib study that evaluated how the blockade of *IL-7* would affect immune cells and relevant clinical responses in patients with type 1 diabetes (33). Furthermore, headache was reported by 5 of the 18 healthy volunteers in a study that investigated the safety of GSK2618960, an IL‐7 receptor‐α subunit (CD127) monoclonal antibody (34), suggesting that dysregulation in the *IL-7* levels could be associated with headache.

The T-cell marker gene CD3E was associated with myalgia and chills in our analysis. Curiously, chills was one of the most common adverse events in participants with cutaneous T-cell lymphoma who received the Resimmune, which is a second-generation recombinant immunotoxin composed of the catalytic and translocation domains of diphtheria toxin fused to two single-chain antibody fragments reactive with the extracellular domain of CD3ϵ (35). While no direct association between CD3E gene and myalgia has been found, there is a strong indication that T cells play a key role not only in the induction but also in the suppression of pain (36, 37).

The effectiveness of rVSVΔG-ZEBOV-GP (Ervebo®) has been demonstrated in clinical studies conducted on 15,399 adults in Europe (11), Africa (AGNANDJI) (12, 13) and North America (16). In these populations, the vaccine proved to be safe and induced higher antibody titers sustained for at least 2 years in both European and African vaccines (3), but it showed transient reactogenicity (11). For this and other vaccines with similar adverse reactions, such as the ones against COVID-19 (38–41), the reactogenicity did not prevent the approval of this vaccine, since the benefits highly overcome the risks (15). Nevertheless, more studies investigating the baseline signature of vaccine-induced reactogenicity are necessary for paving the way towards precision vaccinology. This will enable us to identify who will benefit the most and who will be more vulnerable to post-immunization adverse reactions.

## Data availability statement

The original contributions presented in the study are included in the article/**Supplementary Material**, further inquiries can be directed to the corresponding author.

## Ethics statement

The trial protocols were reviewed and approved by the WHO’s Ethics Committee as well as by local ethics committees (USA trial: the Chesapeake Institutional Review Boards (Columbia, MD, USA) and the Crescent City Institutional Review Board (New Orleans, LA, USA); Geneva trial: the Geneva Cantonal Ethics Commission and the Swiss Agency for Therapeutic Products (Swissmedic); Lambaréné trial: the Scientific Review Committee of Centre de Recherches Médicales de Lambaréné (CERMEL), the Institutional Ethics Committee of CERMEL, the National Ethics Committee of Gabon, and the Institutional Ethics Committee of the Universitätsklinikum Tübingen; Kilifi trial: Kilifi Ethics Committee). The studies were conducted in accordance with the local legislation and institutional requirements. The participants provided their written informed consent to participate in this study.

## Author contributions

PG-D: Conceptualization, Data curation, Formal Analysis, Investigation, Methodology, Software, Supervision, Validation, Visualization, Writing – original draft, Writing – review & editing. TD: Data curation, Formal Analysis, Methodology, Visualization, Writing – review & editing. LM: Formal Analysis, Software, Validation, Visualization, Writing – review & editing. IM: Formal Analysis, Software, Validation, Visualization, Writing – review & editing. AN: Formal Analysis, Software, Validation, Visualization, Writing – review & editing. AC: Data curation, Formal Analysis, Visualization, Writing – review & editing. SS: Data curation, Formal Analysis, Visualization, Writing – original draft, Writing – review & editing. AH: Conceptualization, Investigation, Writing – review & editing. DF: Investigation, Writing – review & editing, Resources. EV: Data curation, Writing – review & editing. MH: Data curation, Writing – review & editing. TO: Data curation, Writing – review & editing. FS: Data curation, Writing – review & editing. PM-M: Data curation, Writing – review & editing. AH: Data curation, Investigation, Writing – review & editing. C-AS: Funding acquisition, Project administration, Supervision, Writing – review & editing. DM: Funding acquisition, Project administration, Supervision, Writing – review & editing. HN: Conceptualization, Data curation, Formal Analysis, Funding acquisition, Investigation, Methodology, Resources, Software, Supervision, Validation, Visualization, Writing – original draft.

## Group members of the VSV-EBOVAC consortium

Selidji T. Agnandji, Rafi Ahmed, Jenna Anderson, Floriane Auderset, Philip Bejon, Luisa Borgianni, Jessica Brosnahan, Annalisa Ciabattini, Olivier Engler, Mariëlle C. Haks, Ali M. Harandi, Donald Gray Heppner, Alice Gerlini, Angela Huttner, Peter G. Kremsner, Donata Medaglini, Thomas P. Monath, Francis M. Ndungu, Patricia Njuguna, Tom H. M. Ottenhoff, David Pejoski, Mark Page, Gianni Pozzi, Francesco Santoro, and Claire-Anne Siegrist.

## Group members of the VSV-EBOPLUS consortium

Selidji T. Agnandji, Luisa Borgianni, Annalisa Ciabattini, Sheri Dubey, Michael Eichberg, Olivier Engler, Alice Gerlini, Patricia Conceição Gonzalez Dias Carvalho, Mariëlle C. Haks, Ali M. Harandi, Angela Huttner, Peter G. Kremsner, Kabwende Lumeka, Donata Medaglini, Helder I. Nakaya, Sravya S. Nakka, Essone P. Ndong, Tom H. M. Ottenhoff, Gianni Pozzi, Sylvia Rothenberger, Francesco Santoro, Claire-Anne Siegrist, Suzanne van Veen, Eleonora Vianello.
