# Supplementary material for: Baseline gene signatures of reactogenicity to Ebola vaccination: a machine learning approach across multiple cohorts
Source: Front Immunol. 2023 Nov 8;14:1259197. doi: 10.3389/fimmu.2023.1259197 (PMC10663260; doi:10.3389/fimmu.2023.1259197)
Supplement: Supplementary file 1 [file Image_1.pdf]

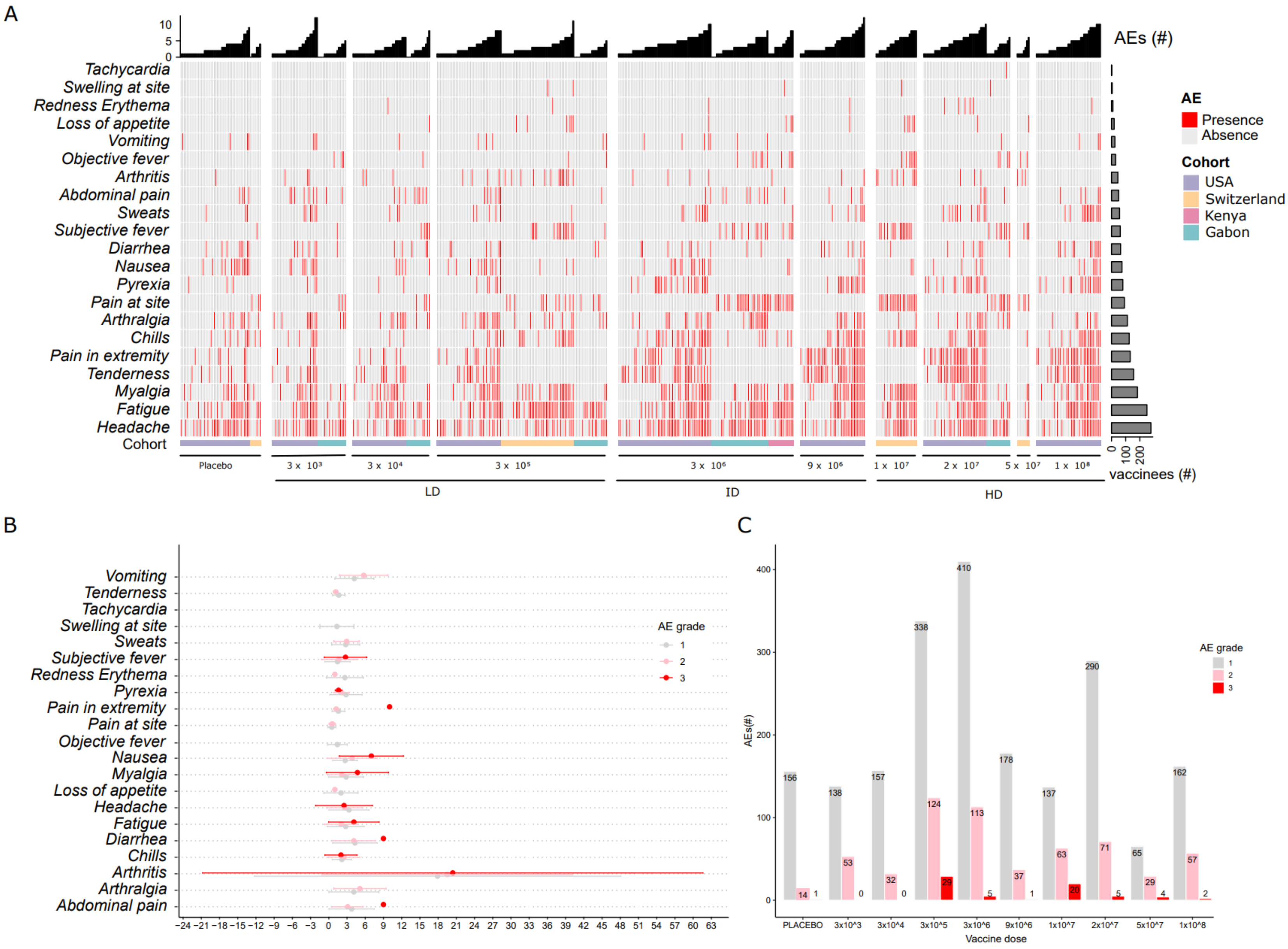
A
B
C
AEs (#)
10
5
0
Tachycardia
Swelling at site
Redness Erythema
Loss of appetite
Vomiting
Objective fever
Arthritis
Abdominal pain
Sweats
Subjective fever
Diarrhea
Nausea
Pyrexia
Pain at site
Arthralgia
Chills
Pain in extremity
Tenderness
Myalgia
Fatigue
Headache
Cohort
Placebo
3 x 10³
3 x 10⁴
3 x 10⁵
3 x 10⁶
9 x 10⁶
1 x 10⁷
2 x 10⁷
5 x 10⁷
1 x 10⁸
LD
ID
HD
0
100
200
vaccinees (#)
AE
Presence
Absence
Cohort
USA
Switzerland
Kenya
Gabon
Vomiting
Tenderness
Tachycardia
Swelling at site
Sweats
Subjective fever
Redness Erythema
Pyrexia
Pain in extremity
Pain at site
Objective fever
Nausea
Myalgia
Loss of appetite
Headache
Fatigue
Diarrhea
Chills
Arthritis
Arthralgia
Abdominal pain
AE grade
1
2
3
−24 −21 −18 −15 −12 -9 -6 -3 0 3 6 9 12 15 18 21 24 27 30 33 36 39 42 45 48 51 54 57 60 63
AEs(#)
400
300
200
100
0
156 14 1
138 53 0
157 32 0
338 124 29
410 113 5
178 37 1
137 63 20
290 71 5
65 29 4
162 57 2
PLACEBO
3x10^3
3x10^4
3x10^5
3x10^6
9x10^6
1x10^7
2x10^7
5x10^7
1x10^8
Vaccine dose
AE grade
1
2
3

**Supplementary Figure 1:** Complete Adverse Events description for the 4 cohorts. **(A)** Heatmap showing the presence (red) and absence (light grey) of all vaccine-related adverse events. The most frequent AEs are shown at the bottom of the heatmap, and the columns are ordered per dose and cohort, as shown in the bottom annotation (USA, purple; Switzerland, yellow; Gabon, green and Kenya, pink). The number of AEs per participant is shown in the bar plot at the top and the total number of reported adverse events is shown in the bar plot on the right. **(B)** Dot plot showing the mean and the standard deviation of the onset day. The colours represent the AE grade: grade 1 (grey), grade 2 (pink) and grade 3 (red). **(C)** The bar plot shows the total number of adverse events per dose and per AE grade (1 - grey; 2 - pink and 3 - red). All participants who received the vaccine with available dcRT-MLPA data at day 0 (*n* = 343) were included; they had received different doses of rVSVΔG-ZEBOV-GP, distributed as follows: $1x10^{8}$ pfu (US n = 35), $5x10^{7}$ pfu (Switzerland n = 16), $2x10^{7}$ pfu (US n = 35, Gabon n = 20, Kenya n = 20) , $1x10^{7}$ pfu (Switzerland n = 35), $9x10^{6}$ pfu (USA n = 35), $3x10^{6}$ pfu (USA n = 20, Gabon n = 35, Kenya n = 20), $3x10^{5}$ pfu (Switzerland n = 51, Gabon n = 21 ), $3x10^{4}$ pfu (Gabon n = 19), $3x10^{3}$ pfu (Gabon n = 21 ) and placebo (Switzerland n=13, USA n=19
