# Supplementary material for: Baseline gene signatures of reactogenicity to Ebola vaccination: a machine learning approach across multiple cohorts
Source: Front Immunol. 2023 Nov 8;14:1259197. doi: 10.3389/fimmu.2023.1259197 (PMC10663260; doi:10.3389/fimmu.2023.1259197)
Supplement: Supplementary file 2 [file Image_2.pdf]

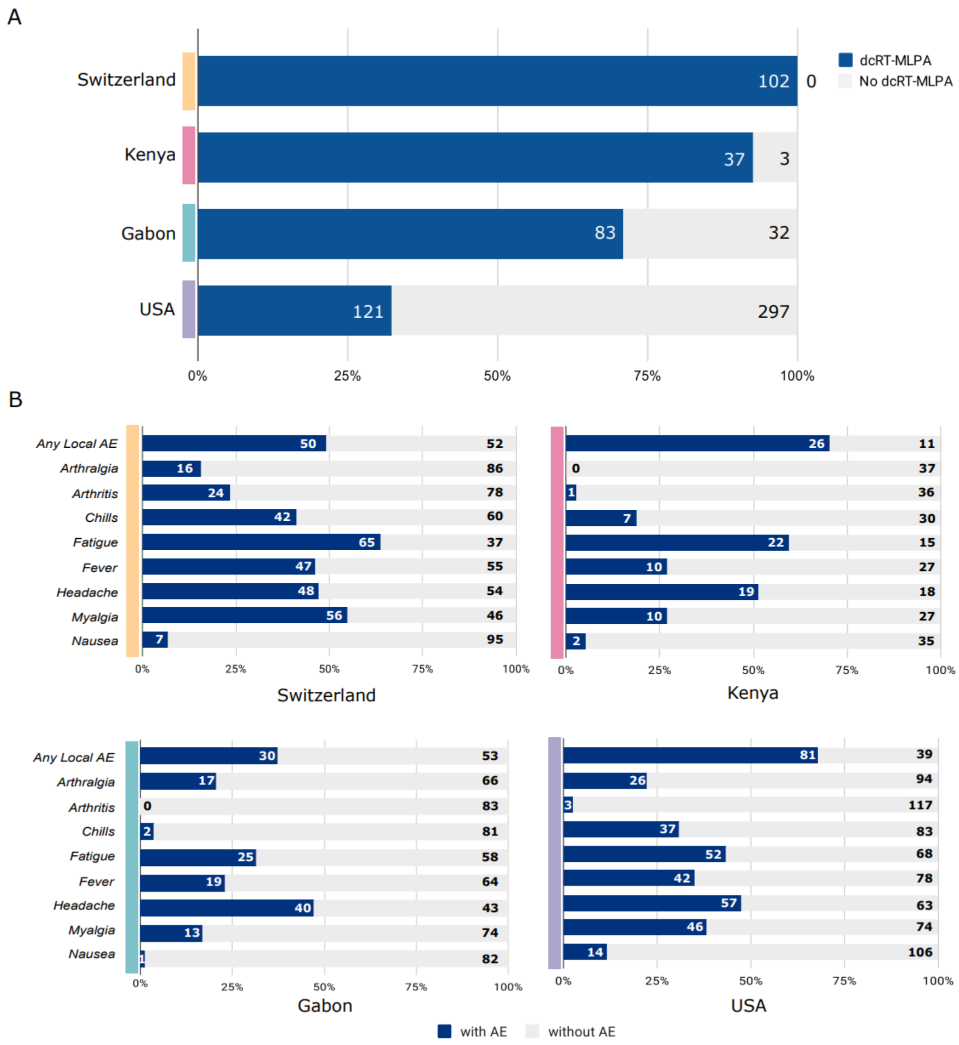

Supplementary Figure 2. dcRT-MLPA availability data description. **(A)** Absolute number and proportion of participants with available dcRT-MLPA data (blue) or not (light gray) for each cohort. **(B)** Frequency of presence (blue) or absence (gray) of adverse events between volunteers with dcRT-MLPA data available.
