# Supplementary material for: Baseline gene signatures of reactogenicity to Ebola vaccination: a machine learning approach across multiple cohorts
Source: Front Immunol. 2023 Nov 8;14:1259197. doi: 10.3389/fimmu.2023.1259197 (PMC10663260; doi:10.3389/fimmu.2023.1259197)
Supplement: Supplementary file 3 [file Image_3.pdf]

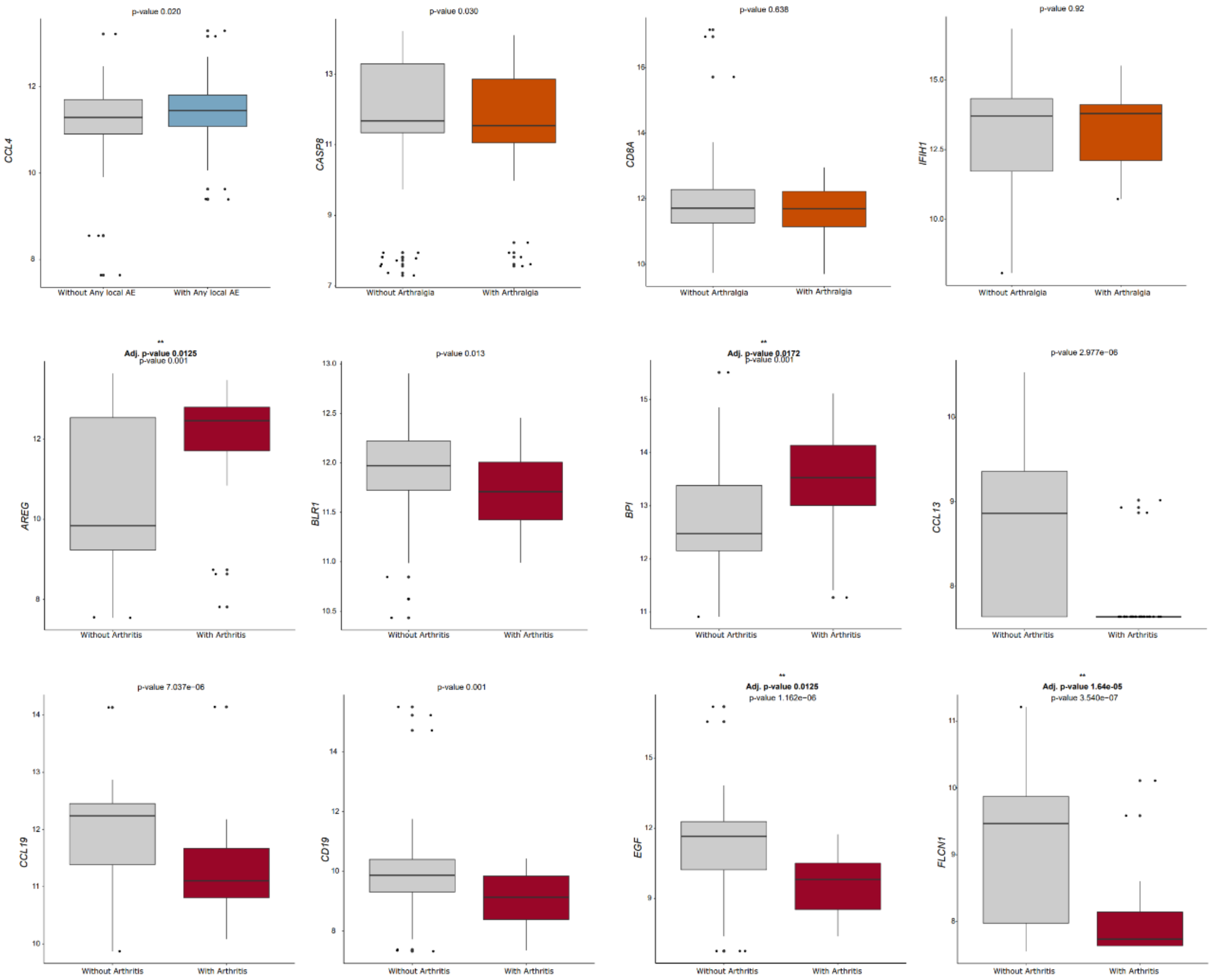
p-value 0.020
CCL4
Without Any local AE
With Any local AE
p-value 0.030
CASP8
Without Arthralgia
With Arthralgia
p-value 0.638
CD8A
Without Arthralgia
With Arthralgia
p-value 0.92
IFIH1
Without Arthralgia
With Arthralgia
**
Adj. p-value 0.0125
p-value 0.001
AREG
Without Arthritis
With Arthritis
p-value 0.013
BLR1
Without Arthritis
With Arthritis
**
Adj. p-value 0.0172
p-value 0.001
BPI
Without Arthritis
With Arthritis
p-value 2.977e-06
CCL13
Without Arthritis
With Arthritis
p-value 7.037e-06
CCL19
Without Arthritis
With Arthritis
p-value 0.001
CD19
Without Arthritis
With Arthritis
**
Adj. p-value 0.0125
p-value 1.162e-06
EGF
Without Arthritis
With Arthritis
**
Adj. p-value 1.64e-05
p-value 3.540e-07
FLCN1
Without Arthritis
With Arthritis

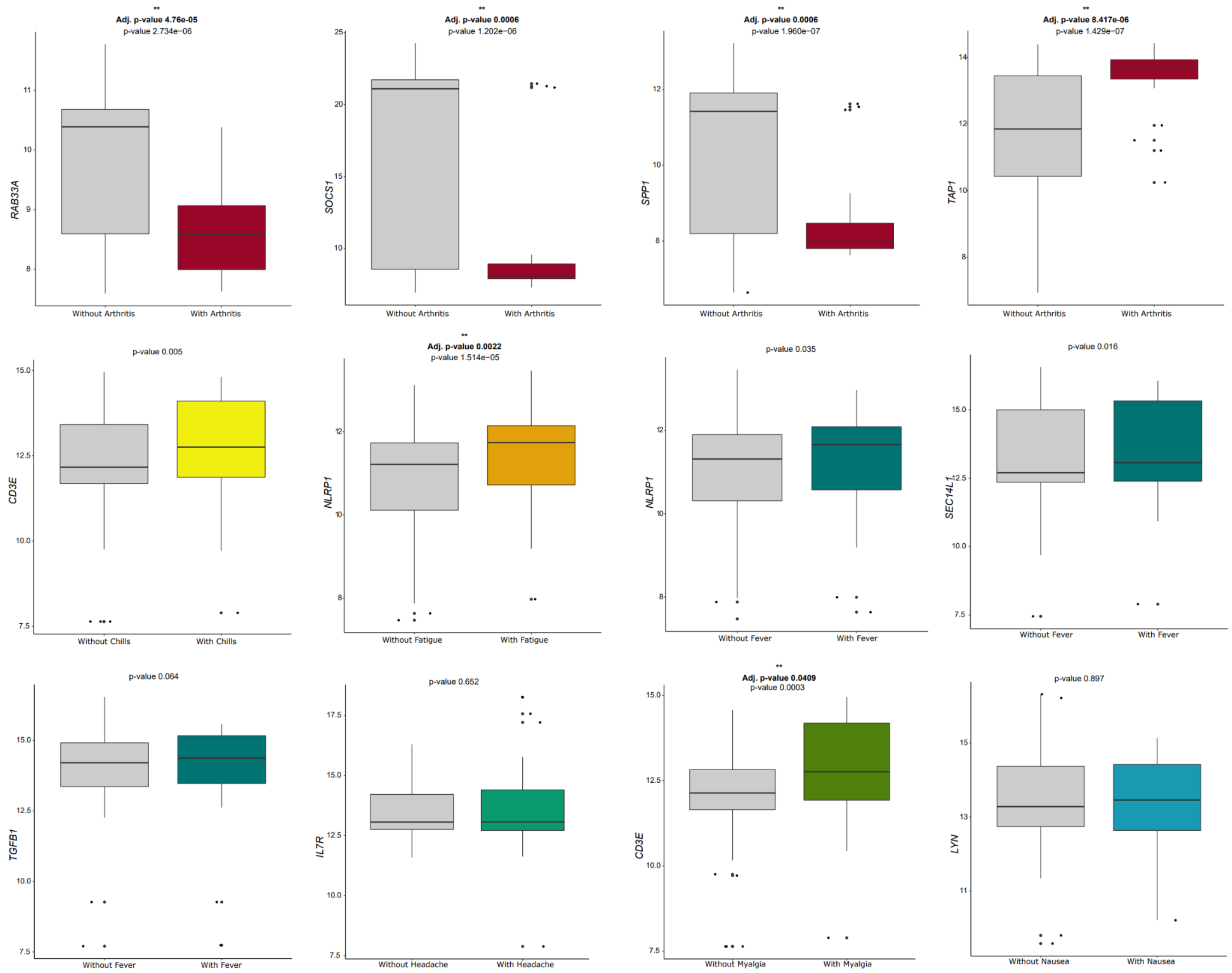

**
Adj. p-value 4.76e-05
p-value 2.734e-06
RAB33A
Without Arthritis
With Arthritis
**
Adj. p-value 0.0006
p-value 1.202e-06
SOCS1
Without Arthritis
With Arthritis
**
Adj. p-value 0.0006
p-value 1.960e-07
SPP1
Without Arthritis
With Arthritis
**
Adj. p-value 8.417e-06
p-value 1.429e-07
TAP1
Without Arthritis
With Arthritis
p-value 0.005
CD3E
Without Chills
With Chills
**
Adj. p-value 0.0022
p-value 1.514e-05
NLRP1
Without Fatigue
With Fatigue
p-value 0.035
NLRP1
Without Fever
With Fever
p-value 0.016
SEC14L1
Without Fever
With Fever
p-value 0.064
TGFB1
Without Fever
With Fever
p-value 0.652
IL7R
Without Headache
With Headache
**
Adj. p-value 0.0409
p-value 0.0003
CD3E
Without Myalgia
With Myalgia
p-value 0.897
LYN
Without Nausea
With Nausea

Supplementary Figure 3. Gene expression dispersion between participants with or without adverse effects. The boxplots show the log2 transformed expression of the genes selected in the network in participants with and without the specific adverse effect. The p-values obtained with the Wilcoxon test are shown in the plots. The significant p-values obtained following Benjamini Hochberg (BH) correction for multiple comparisons are highlighted in bold.
