# Supplementary material for: Baseline gene signatures of reactogenicity to Ebola vaccination: a machine learning approach across multiple cohorts
Source: Front Immunol. 2023 Nov 8;14:1259197. doi: 10.3389/fimmu.2023.1259197 (PMC10663260; doi:10.3389/fimmu.2023.1259197)
Supplement: Supplementary file 4 [file Presentation_1.pdf]

Expanded Methods

Machine-learning-based approach for gene prioritization

Our machine learning approach was developed to select the best features for classifying biological groups. It is divided into three distinct stages: i) feature selection; ii) sorting and; iii) evaluation. Our approach combines different feature selection methods to choose only the most informative features (e.g. Genes, Proteins etc.), then proceeds with ranking the features, assessing their importance for the machine learning model and, finally, evaluating the selected features in the trained models, by using distinct machine learning algorithms.

Feature selection

Feature Selection techniques aim to select a subset of Features of greater relevance for the construction of the predictive model (1). The central premise when using feature selection techniques is that most datasets contain redundant or irrelevant features for the learning of the algorithm and, therefore, can be removed without leading to loss of information in the model (2). This provides benefits such as the reduction of overfitting and training time, as well as an increased accuracy of the model (1, 2).

Our method uses three Feature Selection algorithms:

I. Pearson correlation: verifies the absolute value of the Person correlation between the response variable and the numerical features of the data set (3). In our method we have established an N number of features with the highest correlation;

II. kBest: selects resources according to the highest scoring k (4). In our method the amount of Features selected corresponds to the number N established;

III. Recursive Feature Elimination (RFE): selects features recursively considering sets of Features increasingly smaller. First, an estimator is trained in the initial set of features and the importance of each feature is obtained. Here we used the Support Vector Regression. Then, the less important features are eliminated from the current set (5). The procedure is recursively repeated in the obtained set until the N number of features is reached.

After execution, each feature selection technique provides a list of features of greater relevance according to the employed methodology. From this, a single list is generated with the intersection of features present in at least two of the three techniques. It is important to highlight that, for the calculation of the N number of features used in our approach, the total number of features contained in the single list after the execution of the features intersection is considered. The calculation of the number N is obtained by the following equation:

$$\frac{N}{F} \geq 0.5$$

NF represents the number of features from the single list and N is the value selected from the feature selection techniques. Initially, the number N receives the value 100, being incremented with 100 until the condition of equation 1 is satisfied.

Ordination

In decision trees, each node is a condition of how to divide values into a single resource. Such a condition is based on impurity, which in the case of classification problems is entropy and, for regression problems, is variance. In this sense, when training a decision tree, it is possible to calculate how much each resource contributes to reduce the weighted impurity (6). The ordering step of our machine-learning based approach is based on this logic, and for the calculation of feature importance, the Random Forest algorithm is used, which uses the average of the decrease of impurity on the trees. Thus, after the feature selection step, the most relevant features of the database are ordered according to their importance. It's important to note that during this ordering stage, features with zero importance values are eliminated from the final list of features.

Evaluation

Our method uses different algorithms to evaluate the quality of the chosen features. These algorithms were selected due to the great diversity of their components, which means that they have different methodologies, and they use different mathematical approaches to learn and classify the samples. In this way, it is possible to define a more generalized machine learning model. The algorithms chosen are:

I. Support Vector Machine (SVM): establishes a hyperplane in an N-dimensional space (N - number of resources) that distinctly sorts data points (7).

II. k-Nearest Neighbors (kNN): uses the proximity of the data to perform the classification/prediction on the grouping of an individual data point (8).

III. Naive Bayes: uses the probabilistic paradigm to perform classification tasks, based on the Bayes theorem (9).

IV. AdaBoost Classifier: uses joint learning methods (meta-learning), using an iterative approach to learn from "weak" classifier errors and turn them into strong classifiers (10).

For the performance analysis of the selected algorithms, the methodology of Experimental Planning and Evaluation (11) is used. Moreover, in our approach, a k-fold cross-validation is used, with k = 10, being k-1 for training and the remaining for testing. Thus, it is possible to measure the error estimate more accurately, since the average value estimate tends to a real zero error rate as it increases n, which is usually the case for small sets of examples (11).

Finally, the evaluation of each algorithm is assessed by four commonly used classification metrics: Area Under the ROC Curve (AUC), Precision, Accuracy, and the F1-score, which is a combination of precision and recall metrics. The final output of the model includes the value of each metric for each algorithm evaluated and the final mean and harmonic mean of these values.

Permutation test

We determined the number of features prioritized by our machine learning-based gene ranking tool. To assess the significance of this ranking, we employed the 'random.shuffle' function in Python. This function randomly selects the same number of features as identified by our method, ensuring an unbiased and randomized selection from the dataset. Subsequently, we conducted training and evaluation on this shuffled dataset, following the same methodology as our approach for comparison. This 'shuffling' and 'sorting,' along with the training and evaluation, were repeated ten times, and the results represent the average of these ten repetitions. For instance, if our method selects 100 features, the algorithm randomly 'shuffles' and 'sorts' 100 features from the entire dataset. It then undergoes training and evaluation ten times, calculating average evaluation metrics such as accuracy, precision, F1 score, and AUC. This average serves as the benchmark for comparing the metrics obtained using our prioritization method. Following this process, we compare the metrics of the models generated using the features selected through our method with those obtained using randomly selected features.

Expanded method’s bibliography.

1. Zebari, R., Abdulazeez, A., Zeebaree, D., Zebari, D., & Saeed, J. (2020). A comprehensive review of dimensionality reduction techniques for feature selection and feature extraction. Journal of Applied Science and Technology Trends, 1(2), 56-70.

2. Zhu, Y., Ma, J., Yuan, C., & Zhu, X. (2022). Interpretable learning based dynamic graph convolutional networks for Alzheimer's disease analysis. Information Fusion, 77, 53-61.

3. Liu, Y., Mu, Y., Chen, K., Li, Y., & Guo, J. (2020). Daily activity feature selection in smart homes based on pearson correlation coefficient. Neural Processing Letters, 51(2), 1771-1787.

4. Dissanayake, K., & Md Johar, M. G. (2021). Comparative Study on Heart Disease Prediction Using Feature Selection Techniques on Classification Algorithms. Applied Computational Intelligence and Soft Computing, 2021.

5. Han, Y., Huang, L., & Zhou, F. (2021). A dynamic recursive feature elimination framework (dRFE) to further refine a set of OMIC biomarkers. Bioinformatics, 37(15), 2183-2189.

6. Hasanin, T., Khoshgoftaar, T. M., Leevy, J., & Seliya, N. (2019, April). Investigating random undersampling and feature selection on bioinformatics big data. In 2019 IEEE Fifth International Conference on Big Data Computing Service and Applications (BigDataService) (pp. 346-356). IEEE.

7. Cervantes, J., Garcia-Lamont, F., Rodríguez-Mazahua, L., & Lopez, A. (2020). A comprehensive survey on support vector machine classification: Applications, challenges and trends. Neurocomputing, 408, 189-215.

8. Tabares-Soto, R., Orozco-Arias, S., Romero-Cano, V., Bucheli, V. S., Rodríguez-Sotelo, J. L., & Jiménez-Varón, C. F. (2020). A comparative study of machine learning and deep learning algorithms to classify cancer types based on microarray gene expression data. PeerJ Computer Science, 6, e270.

9. Sen, P. C., Hajra, M., & Ghosh, M. (2020). Supervised classification algorithms in machine learning: A survey and review. In Emerging technology in modelling and graphics (pp. 99-111). Springer, Singapore.

10. Almustafa, K. M. (2020). Prediction of heart disease and classifiers' sensitivity analysis. BMC bioinformatics, 21(1), 1-18.

11. Mano, L. Y., Faiçal, B. S., Gonçalves, V. P., Pessin, G., Gomes, P. H., de Carvalho, A. C., & Ueyama, J. (2020). An intelligent and generic approach for detecting human emotions: a case study with facial expressions. Soft Computing, 24(11), 8467-8479.
