# Supplementary material for: Baseline gene signatures of reactogenicity to Ebola vaccination: a machine learning approach across multiple cohorts
Source: Front Immunol. 2023 Nov 8;14:1259197. doi: 10.3389/fimmu.2023.1259197 (PMC10663260; doi:10.3389/fimmu.2023.1259197)
Supplement: Supplementary file 5 [file Table_1.pdf]

Supplementary table 1. The absolute number and percentage of participants' genders within each cohort.

Cohort	Absolute number (Percentage)	
	Male	Female
USA	267 (52,05%)	246 (47,95%)
Switzerland	62 (53,91%)	53 (46,09%)
Kenya	30 (75%)	10 (25%)
Gabon	94 (81,74%)	21 (18,26%)
