# Supplementary material for: Baseline gene signatures of reactogenicity to Ebola vaccination: a machine learning approach across multiple cohorts
Source: Front Immunol. 2023 Nov 8;14:1259197. doi: 10.3389/fimmu.2023.1259197 (PMC10663260; doi:10.3389/fimmu.2023.1259197)
Supplement: Supplementary file 6 [file Table_2.pdf]

Supplementary table 2. The median age for each cohort.

Cohort	Age (Median)
USA	36
Switzerland	41
Kenya	31.5
Gabon	25
