# Supplementary material for: Baseline gene signatures of reactogenicity to Ebola vaccination: a machine learning approach across multiple cohorts
Source: Front Immunol. 2023 Nov 8;14:1259197. doi: 10.3389/fimmu.2023.1259197 (PMC10663260; doi:10.3389/fimmu.2023.1259197)
Supplement: Supplementary file 7 [file Table_3.pdf]

**Supplementary table 3.** The percentage of participants with dcRT-MLPA data available within each cohort.

| Cohort | dcRT-MLPA data available (%) |
|---|---|
| Switzerland | 100 |
| Kenya | 92.5 |
| Gabon | 72.17 |
| USA | 28.95 |
