# Supplementary material for: Baseline gene signatures of reactogenicity to Ebola vaccination: a machine learning approach across multiple cohorts
Source: Front Immunol. 2023 Nov 8;14:1259197. doi: 10.3389/fimmu.2023.1259197 (PMC10663260; doi:10.3389/fimmu.2023.1259197)
Supplement: Supplementary file 8 [file Table_4.pdf]

Supplementary table 4. Comparison of the ranking and frequency of adverse effects between 786 participants with reactogenicity data and 343 participants with available dcRT-MLPA data.

	786 volunteers		343 dcRT-MLPA volunteers	
**AEs**	**Ranking**	**AEs frequency (%)**	**Ranking**	**AEs frequency (%)**
Any Local AE	1	53.25	1	48.4
Arthralgia	7	13.67	7	17.2
Arthritis	9	6.39	8	8.2
Chills	6	21.5	6	29.9
Fatigue	2	49.17	2	48.1
Fever	5	28.9	5	34.4
Headache	3	46.55	3	47.5
Myalgia	4	31.27	4	36.7
Nausea	8	6.3	9	7
