# Supplementary material for: Baseline gene signatures of reactogenicity to Ebola vaccination: a machine learning approach across multiple cohorts
Source: Front Immunol. 2023 Nov 8;14:1259197. doi: 10.3389/fimmu.2023.1259197 (PMC10663260; doi:10.3389/fimmu.2023.1259197)
Supplement: Supplementary file 9 [file Table_5.pdf]

Supplementary table 5. List of the 144 genes and their associated pathways that are part of the dcRT-MLPA panel.

Gene	Pathway
AIRE	T cell subset markers
AREG	Cell growth - proliferation
ASAP1	Small GTPases - (Rho) GTPase activating proteins
BCL2	Apoptosis - Survival
BLR1	G protein-coupled receptors
BMP6	Cell growth - proliferation
BPI	Anti-microbial activity
CAMTA1	Transcriptional regulators - activators
CASP8	Apoptosis - Survival
CCL11	Chemokines
CCL13	Chemokines
CCL19	Chemokines
CCL2	Myeloid associated genes
CCL22	Myeloid associated genes
CCL3	Myeloid associated genes
CCL4	Treg associated genes
CCL5	Myeloid associated genes
CCR7	T cell subset markers
CD14	Myeloid associated genes
CD163	Myeloid associated genes
CD19	Immune cell subset markers - B cells
CD209	Pattern recognition receptors
CD274	IFN signaling genes
CD3E	T cell subset markers
CD4	T cell subset markers
CD8A	T cell subset markers
CLEC7A	Pattern recognition receptors
CTLA4	Treg associated genes
CX3CL1	Chemokines

CXCL10	Th1 associated genes
CXCL13	Myeloid associated genes
CXCL9	Chemokines
DSE	Inflammation
EGF	Cell growth - proliferation
FASLG	Apoptosis - Survival
FCGR1A	IFN signaling genes
FLCN1	Apoptosis - Survival
FOXP3	Treg associated genes
FPR1	Myeloid associated genes
GATA3	Th2 associated genes
GBP1	IFN signaling genes
GBP2	IFN signaling genes
GBP5	IFN signaling genes
GNLY	Cytotoxicity markers
GZMA	Cytotoxicity markers
GZMB	Cytotoxicity markers
HCK	Cell activation
HPRT	Mitochondrial Stress - Proteasome
IFI16	IFN signaling genes
IFI35	IFN signaling genes
IFI44	IFN signaling genes
IFI44L	IFN signaling genes
IFI6	IFN signaling genes
IFIH1	IFN signaling genes
IFIT2	IFN signaling genes
IFIT3	IFN signaling genes
IFIT5	IFN signaling genes
IFITM3	IFN signaling genes
IFNG	Th1 associated genes
IL10	Th2 associated genes
IL12A	Myeloid associated genes
IL12B	Myeloid associated genes
IL13	Th2 associated genes

IL15	Th1 associated genes
IL17A	Th17 associated genes
IL1B	Th1 associated genes
IL2	Th1 associated genes
IL22RA1	Th17 associated genes
IL23A	Myeloid associated genes
IL2RA	Treg associated genes
IL4	Th2 associated genes
IL4d2	Th2 associated genes
IL5	Th2 associated genes
IL6	Th2 associated genes
IL7R	T cell subset markers
IL9	Th9 associated genes
INDO	IFN signaling genes
IRF7	IFN signaling genes
KIF1B	Intracellular transport
LAG3	Treg associated genes
LTF	Anti-microbial activity
LYN	Cell activation
MARCO	Scavenger receptors
MMP9	Inflammation
MRC1	Pattern recognition receptors
MRC2	Pattern recognition receptors
NCAM1	Immune cell subset markers - NK cells
NEDD4L	E3 ubiquitin protein ligases
NLRC4	Inflammasome components
NLRP1	Inflammasome components
NLRP10	Inflammasome components
NLRP11	Inflammasome components
NLRP12	Inflammasome components
NLRP13	Inflammasome components
NLRP2	Inflammasome components
NLRP3	Inflammasome components
NLRP4	Inflammasome components

NLRP6	Inflammasome components
NLRP7	Inflammasome components
NOD1	Pattern recognition receptors
NOD2	Pattern recognition receptors
OAS1	IFN signaling genes
OAS2	IFN signaling genes
OAS3	IFN signaling genes
PRF1	Cytotoxicity markers
PTPRCv1	T cell subset markers
PTPRCv2	T cell subset markers
RAB13	Small GTPases - (Rho) GTPase activating proteins
RAB24	Small GTPases - (Rho) GTPase activating proteins
RAB33A	Small GTPases - (Rho) GTPase activating proteins
RORC	Th17 associated genes
SEC14L1	Intracellular transport
SLAMF7	Cell activation
SOCS1	IFN signaling genes
SPP1	Inflammation
STAT1	IFN signaling genes
STAT2	IFN signaling genes
TAGAP	Small GTPases - (Rho) GTPase activating proteins
TAP1	IFN signaling genes
TAP2	IFN signaling genes
TBC1D7	Small GTPases - (Rho) GTPase activating proteins
TBX21	Th1 associated genes
TGFB1	Treg associated genes
TGFBR2	Cell growth - proliferation
TIMP2	Inflammation
TLR1	Pattern recognition receptors
TLR10	Pattern recognition receptors
TLR2	Pattern recognition receptors
TLR3	Pattern recognition receptors

TLR4	Pattern recognition receptors
TLR5	Pattern recognition receptors
TLR6	Pattern recognition receptors
TLR7	Pattern recognition receptors
TLR8	Pattern recognition receptors
TLR9	Pattern recognition receptors
TNF	Th1 associated genes
TNFRSF18	Treg associated genes
TNFRSF1A	Apoptosis - Survival
TNFRSF1B	Apoptosis - Survival
TNIP1	Inflammation
TWIST1	Transcriptional regulators - activators
VEGF	Cell growth - proliferation
ZNF331	Transcriptional regulators - activators
ZNF532	Transcriptional regulators - activators
